# Spatio-Temporal Factors Associated with Meningococcal Meningitis Annual Incidence at the Health Centre Level in Niger, 2004–2010

**DOI:** 10.1371/journal.pntd.0002899

**Published:** 2014-05-22

**Authors:** Juliette Paireau, Halima B. Maïnassara, Jean-François Jusot, Jean-Marc Collard, Issa Idi, Jean-Paul Moulia-Pelat, Judith E. Mueller, Arnaud Fontanet

**Affiliations:** 1 Unité d'Epidémiologie des Maladies Emergentes, Institut Pasteur, Paris, France; 2 Univ. Pierre et Marie Curie, Cellule Pasteur UPMC, Paris, France; 3 Unité d'Epidémiologie/Santé-Environnement-Climat, Centre de Recherche Médicale et Sanitaire (CERMES)/Réseau International des Instituts Pasteur, Niamey, Niger; 4 Unité de Biologie, Centre de Recherche Médicale et Sanitaire (CERMES)/Réseau International des Instituts Pasteur, Niamey, Niger; 5 EHESP French School of Public Health, Sorbonne Paris Cité, Rennes, France; 6 Conservatoire National des Arts et Métiers, Chaire Santé et Développement, Paris, France; Institute of Collective Health, Federal University of Bahia, Brazil

## Abstract

**Background:**

Epidemics of meningococcal meningitis (MM) recurrently strike the African Meningitis Belt. This study aimed at investigating factors, still poorly understood, that influence annual incidence of MM serogroup A, the main etiologic agent over 2004–2010, at a fine spatial scale in Niger.

**Methodology/Principal Findings:**

To take into account data dependencies over space and time and control for unobserved confounding factors, we developed an explanatory Bayesian hierarchical model over 2004–2010 at the health centre catchment area (HCCA) level. The multivariate model revealed that both climatic and non-climatic factors were important for explaining spatio-temporal variations in incidence: mean relative humidity during November–June over the study region (posterior mean Incidence Rate Ratio (IRR) = 0.656, 95% Credible Interval (CI) 0.405–0.949) and occurrence of early rains in March in a HCCA (IRR = 0.353, 95% CI 0.239–0.502) were protective factors; a higher risk was associated with the percentage of neighbouring HCCAs having at least one MM A case during the same year (IRR = 2.365, 95% CI 2.078–2.695), the presence of a road crossing the HCCA (IRR = 1.743, 95% CI 1.173–2.474) and the occurrence of cases before 31 December in a HCCA (IRR = 6.801, 95% CI 4.004–10.910). At the study region level, higher annual incidence correlated with greater geographic spread and, to a lesser extent, with higher intensity of localized outbreaks.

**Conclusions:**

Based on these findings, we hypothesize that spatio-temporal variability of MM A incidence between years and HCCAs result from variations in the intensity or duration of the dry season climatic effects on disease risk, and is further impacted by factors of spatial contacts, representing facilitated pathogen transmission. Additional unexplained factors may contribute to the observed incidence patterns and should be further investigated.

## Author Summary

Meningococcal meningitis (MM) is a severe infection of the meninges caused by a bacterium transmitted through respiratory droplets. During January–May, epidemics of MM recurrently strike sub-Saharan countries, including Niger. Understanding why epidemics occur in a particular place at a particular time would help public health authorities to develop more efficient prevention strategies. To date, factors that govern the occurrence of localized outbreaks are still poorly understood and epidemics remain unpredictable. In this retrospective study (2004–2010), we developed a statistical model in order to investigate the influence of various factors (climatic, demographic, epidemiologic, etc.) on the annual incidence of MM serogroup A at a fine spatial scale (the health centre catchment area) in Niger. We found that mean relative humidity and occurrence of early rains were protective climatic factors and that a higher risk was associated with the presence of a road, the percentage of neighbouring areas having cases and the occurrence of early cases before January. These findings contribute to improve our understanding of MM epidemics in Africa and the associated factors, and might be used in the future for the subsequent development of an early warning system.

## Introduction

Meningococcal meningitis (MM) is caused by *Neisseria meningitidis* (*Nm*), a commensal bacterium of the human nasopharynx transmitted by direct contact with respiratory droplets from carriers and causing meningitis after crossing the nasopharyngeal mucosa. Epidemics of meningococcal meningitis recurrently strike countries of the African Meningitis Belt [1]. In this sub-Saharan area, MM dynamics is characterized by seasonality and spatio-temporal heterogeneity: the disease is endemic all year round but every dry season, a hyper-endemic or epidemic increase in incidence is observed, the magnitude of which varies between years and regions [2], [3]. Within a country, localized outbreaks are reported at sub-district scales [4]–[6]. Most epidemics have been caused by meningococci of serogroup A but C, W or X outbreaks have also been reported [7]–[9]. Niger, a landlocked country of the Belt, reported between 1000 and 13500 suspected meningitis cases annually during 2004–2010, with case-fatality rates of 4–12% [10]. Over the study period (2004–2010), the surveillance-based control strategy applied in Niger was to launch reactive vaccination campaigns with A/C or A/C/W polysaccharide vaccines once an outbreak has exceeded a threshold defined at the district level by the World Health Organization (WHO) [11].

More than 100 years after the first major epidemic reported in the Belt, the reasons for the peculiar epidemiology of MM in Africa are still poorly understood [12]. A combination of concomitant factors is probably necessary to trigger an epidemic in a particular place at a particular time, involving the organism (e.g. strain virulence and transmissibility [13]), the host (e.g. immune status and susceptibility [14], [15]) and the environment (e.g. dry climate and dusty winds [16]).

Previous statistical ecologic studies aiming at explaining the spatio-temporal dynamics of MM epidemics in the Belt were mainly focused on climatic risk factors. These studies sought for drivers of either the seasonality of epidemics (i.e. when the onset/peak/end of the meningitis season occur) or their intensity (i.e. magnitude of incidence over a chosen time period) at different spatial scales. According to most authors, the temporal dynamics of sub-Saharan climate is the major driver of MM seasonality in the Belt [2], [3], [17]–[19]. The suspected contribution of climatic factors to the intensity of epidemics is still under debate. At the country level, Yaka et al partly related annual incidence in Niger to the northern component of wind during November to December [20]. At the district level, annual incidence in four African countries was correlated to rainfall amount and dust load in the pre-, post- and epidemic season [21] and monthly incidence in one district of Ghana was modelled by a combination of various climatic and non-climatic variables [22].

However, to our best knowledge, none of the published statistical models tried to explain intensity of meningitis outbreaks at a finer spatial scale than the district, whereas recent studies in Niger and Burkina Faso demonstrated that outbreaks occur at sub-district scales in highly localized clusters [4]–[6]. Besides, whereas two neighbouring areas (sharing similar climatic conditions) can have different epidemic behaviours [4], [6], few models combined climatic factors with other types of risk factors suspected to interact, such as previous epidemics, vaccination campaigns, population density or proximity to infected regions.

The objective of our paper was therefore to study the influence of climatic and non-climatic factors on the spatio-temporal variations of annual incidence of MM serogroup A, the main etiologic agent over the study period, at the health centre catchment area (HCCA) scale in Niger, using a database of laboratory-confirmed cases and developing an explanatory Bayesian hierarchical model from 2004 to 2010 at the HCCA-year level.

## Methods

### Ethics statement

This study was approved by the Clinical Research Committee of Institut Pasteur and authorized by the National Consultative Ethics Committee of Niger and the two French data protection competent authorities: CCTIRS (Comité Consultatif sur le Traitement de l'Information en matière de Recherche dans le domaine de la Santé) and CNIL (Commission Nationale de l'Informatique et des Libertés). The data collected involving patients were anonymized.

### Cartographic data

Spatial analyses were based on the National Health Map of Niger, created by the Centre de Recherche Médicale et Sanitaire (CERMES) in 2008, at the level of the HCCAs, areas which include all villages served by the same integrated health centre. We used the 2010 updated version of this National Health Map of 732 HCCAs, in the WGS84 geodetic system with UTM 32N projection. On average, a HCCA covers a 40×40 km^2^ area.

### Study region

**Figure 1:**
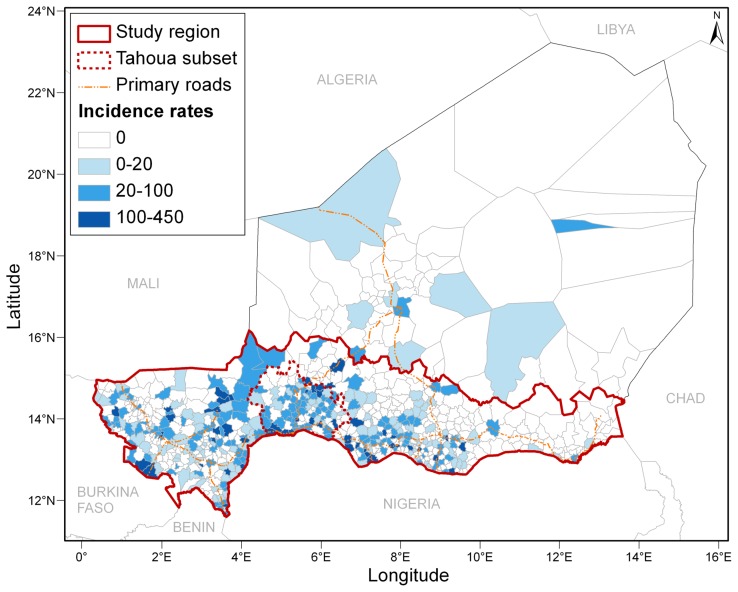
Spatial distribution of meningococcal meningitis A in Niger from July 2003 to June 2010. Cumulative incidence rates in each health centre catchment area (cases per 100000 inhabitants).

The study region comprised the 669 HCCAs of the southern most populated part of Niger, located roughly to the south of the 16^th^ parallel (Figure 1). It represents 96% of the national population of 17 138 707 inhabitants (2012 national census). The semi-arid tropical climate of this Sahelian region is characterized by a long dry season from October to May and a rainy season from June to September. In the North lies the Sahara desert, with less than one inhabitant per km^2^.

### Meningitis data

The CERMES is the national reference laboratory in charge of the microbiological surveillance of bacterial meningitis in Niger. Basically, cerebrospinal fluid (CSF) samples taken from suspected cases of meningitis by health care workers are routinely collected throughout Niger and the etiological diagnosis is carried out by Polymerase Chain Reaction (PCR) for all CSF. This enhanced surveillance system is active in the whole country since 2002, and has been described in detail elsewhere [4]. We used the CERMES database for a retrospective study on confirmed MM A cases between 1 July 2003 and 30 June 2010. We aggregated MM A cases by HCCA and epidemiological year, defined as running from 1 July of the year *n–1* to 30 June of the year *n*, in order to cover an entire meningitis season.

### Vaccination data

Health districts were contacted to obtain data on polysaccharide vaccines (number of delivered vaccine doses and/or vaccine coverage) at the HCCA level over the study period and the previous two years. Full vaccination records could be collected only for Tahoua region over 2002–2010. Missing data in records from other regions did not enable us to use them in our analyses. We thus studied the effect of previous vaccination campaigns conducted in Tahoua region during the years *n-1* and *n-2* on MM incidence of year *n*. We considered different forms for the vaccination covariate: either the coverage rate (as a continuous or categorized variable), the vaccination status (vaccinated: yes/no), or the exceedance of several coverage thresholds (above threshold: yes/no). The cumulative effect of successive vaccination campaigns could not be studied as only one HCCA was vaccinated two years in a row.

### Demographic data

The Institut National de la Statistique (INS) provided the number of inhabitants per village according to the 2001 national census. We aggregated the villages' populations at the HCCA level and applied a mean annual population growth rate of 3.3% (provided by the INS). We computed the population density covariate as the number of inhabitants per HCCA divided by the HCCA surface area calculated in ArcGIS software (version 10.0, ESRI Inc. Redlands, CA).

### Road network

We retrieved a shapefile of primary roads from the HealthMapper application of the WHO. This shapefile was superimposed to the National Health Map in ArcGIS. For each HCCA, we computed its minimum distance to the closest primary road and expressed it as a binary covariate (road *versus* no road) or a categorical covariate (classes of distance).

### Landcover data

The landcover classification for Niger was obtained at a 1 km^2^ resolution, from the Land Cover Map of Africa from the Global Land Cover 2000 Project [23]. The main vegetation types represented in our geographical subset were different classes of shrublands, grasslands and croplands.

### Climate and aerosol data

Gridded climate data from 2003 to 2010 were extracted from ERA-Interim reanalysis, produced by the European Centre for Medium-Range Weather Forecasts (ECMWF) [24]. We retrieved relative humidity, temperature, total precipitation, U (west-east) and V (south-north) wind components at a 0.75° spatial resolution at a daily time-step. To characterize the wind-blown mineral dust emission from the Sahara, we used the Absorbing Aerosol Index (AAI), a dimensionless quantity which indicates the presence of ultraviolet-absorbing aerosols in the Earth's atmosphere [25]. The AAI used in this study is derived from the reflectances measured by SCIAMACHY (Scanning Imaging Absorption Spectrometer for Atmospheric Chartography) satellite instrument in the ultraviolet wavelength range [26]. We retrieved monthly gridded data (1.00°×1.25° latitude-longitude grid) from 2003 to 2010 (www.temis.nl/airpollution/absaai/). As we were interested in how the climate of a given year or season can influence the annual epidemic magnitude, we calculated multi-monthly means of each climatic variable, averaged over periods relevant to the meningitis season or the seasonal cycles of each climatic variable, both for each HCCA and for the whole study region (see Figure S1 and Text S1 for further details).

### Altitude data

Shuttle Radar Topography Mission (SRTM) elevation data were obtained from the processed CGIAR-CSI (Consortium for Spatial Information) SRTM 90 m Digital Elevation Dataset version 4.1 [27], available as 5°×5° tiles at a 3 arc second resolution (approximately 90 m). Six tiles were downloaded and combined in ArcGIS to cover the whole study region.

### Database preparation

Finally, we collated these multi-source and multi-format spatio-temporal datasets and reconciled data at the HCCA level (i.e. cartographic, epidemiological, vaccination and demographic data) and gridded data (i.e. landcover, climate, AAI and altitude data) by averaging the gridded values over each HCCA using the statistical computing software R (version 2.15.3, R Core Team, R Foundation for Statistical Computing, Vienna, Austria). Then, in addition to the covariates described above, we created supplementary variables to include in the statistical analyses. To take into account potential interactions with bordering countries, we calculated in ArcGIS the minimum distance of each HCCA to the closest border and expressed it as a binary variable (border *versus* no border) or a categorical variable (classes of distance and classes of bordering countries). The five bordering countries of our geographical subset are shown in Figure 1. To account for potential geographic disparities in accessibility to health centres, we computed for each HCCA the mean distance (weighted by the villages' population) from villages to their health centre. To represent the tendency of meningitis to occur in spatio-temporal clusters of neighbouring infected HCCAs, we computed “neighbourhood” variables, using various definitions for this spatio-temporal interaction (presence/total number of MM A cases in neighbours, mean/maximum incidence and number/percentage of neighbours with MM A cases, over an epidemiological year). Neighbours were defined as adjacent HCCAs (first order neighbours), since a previous analysis showed that the median size of spatial clusters was of two neighbouring HCCAs [4]. We also computed «historical» variables describing what happened the previous year in terms of presence/number of MM A cases and incidence, in each HCCA, in its neighbours and in its district (upper administrative level) as potential proxies for natural immunity. We computed similar variables for other *Nm* serogroups at the HCCA level in order to explore potential interactions between serogroups. Finally, we included in the analyses the presence of early cases in each HCCA, defined as cases occurring before 31 December following [20], as an early start of the hyper-endemic increase could indicate a higher epidemic risk.

### Statistical analysis

First, for descriptive purposes, we explored whether the annual epidemic magnitude in the study region could be related to the annual and early geographical distribution of MM A cases and localized outbreaks, using Pearson correlation coefficient. We defined localized outbreaks as HCCAs exceeding an annual incidence threshold of 20/100000, corresponding to the 95^th^ percentile of incidence, following the primary reference used in [6].

Then, to investigate the spatio-temporal association of MM A annual incidence at the HCCA level with climatic and non-climatic factors, we developed a retrospective hierarchical model in Niger for 2004–2010, over two geographical subsets: (i) over the whole study region of 669 HCCAs and (ii) over a subset of 95 HCCAs (located in Tahoua region) for which vaccination data were fully available.

The modelling approach we adopted was a Bayesian negative binomial generalized linear mixed model (GLMM). We assumed that the number of observed MM A cases in each HCCA *i* and year *t* followed a negative binomial distribution with an unknown scale parameter *κ* and mean *μ_it_*. We modelled *log(μ_it_)* as a function of covariates as described above and appropriate random effects. Basically, we included spatial random effects at the HCCA level, separated into a spatially unstructured component to capture the influence of unknown factors that are independent across areas and a spatially structured component to capture the influence of spatially correlated effects. The temporal structure was modelled by yearly random intercepts. We included the expected number of cases in each HCCA *i* and year *t* as an offset in the model to estimate the incidence rate ratios (IRRs) associated with a unit increase in exposure, by exponentiating the regression coefficients.

A preliminary forward stepwise covariate selection was performed in R software, estimating parameters by maximum likelihood. The Bayesian multivariate model was subsequently developed in WinBUGS [28], using Markov chain Monte Carlo (MCMC) simulation methods. Further details on the modelling approach are given in Text S2.

## Results

### Description of the data

**Figure 2:**
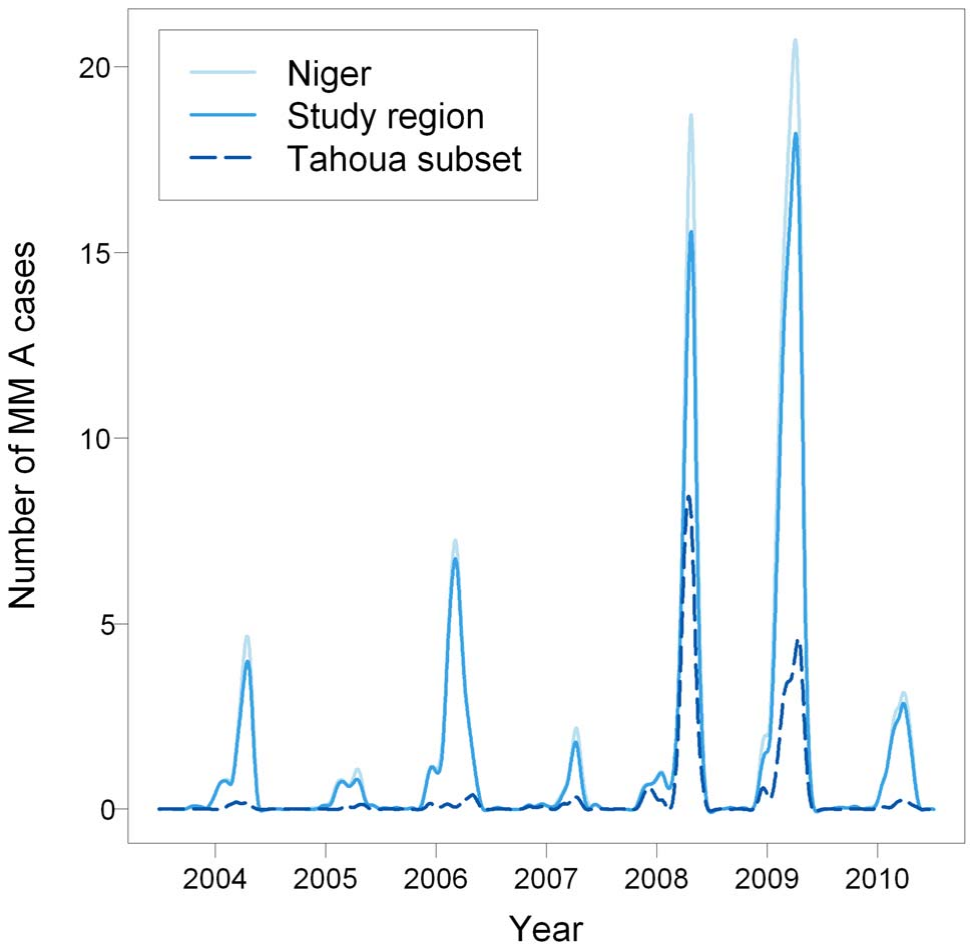
Temporal distribution of meningococcal meningitis (MM) A in Niger from July 2003 to June 2010. Time series of daily cases smoothed by cubic spline.

In Niger, from 1 July 2003 to 30 June 2010, 5512 cases of *Nm* were biologically confirmed. Other aetiologies included *Streptococcus pneumoniae* (N = 850) and *Haemophilus influenzae* (N = 277). Serogroup A accounted for 72.4% (N = 3988) of *Nm* cases and was largely predominant every year, except during 2006 and 2010 when serogroups X and W represented 48.9% and 71.6% of *Nm* cases, respectively. The median age of *Nm* A cases was 8.3 years (interquartile range (IQR) 5–13). Among all *Nm* A cases, 97.0% originated from our study region and 28.0% from our Tahoua subset (Figure 1). *Nm* A cases essentially occurred over a six-month period: 98.1% of them were observed between December and May, with a peak during February–April (80.4%). MM A temporal evolution during July 2003–June 2010 (Figure 2) was characterized by considerable between-year variations (17-fold increase between the lowest annual incidence of 0.7 per 100000 in 2005 and the highest annual incidence of 11.3 per 100000 in 2009).

Among the seven years of the study period, the annual MM A incidence across the whole study region was correlated to the number of HCCAs having at least one MM A case (*r* = 0.95, *p*<0.01), to the number of localized outbreaks (*r* = 0.99, *p*<0.01), to the maximum annual incidence of the localized outbreaks (*r* = 0.80, *p* = 0.03), to the number of HCCAs with at least one early case (*r* = 0.96, *p*<0.01) and to the early incidence across the study region (*r* = 0.93, *p*<0.01). The corresponding graphs are displayed in Figure S2. The median duration of the localized outbreaks (time between first and last cases) was 45 days (IQR 24–75).

### Bayesian multivariate model over the study region

In the Bayesian multivariate model over the whole study region, the overdispersion parameter of the negative binomial (*κ^−1^*) had a posterior mean value of 2.586 (95% CI = 2.223–2.998) (Table 1). This was significantly different from zero, which confirmed that the negative binomial formulation was necessary to account for extra-Poisson variation in the dataset.

**Table 1:** Results from the Bayesian hierarchical model of meningococcal meningitis (MM) A annual incidence at the health centre catchment area (HCCA) level over the study region, Niger 2004–2010.

	Null Model		Multivariate model	
Parameters	Posterior mean	95% CI*	Posterior mean	95% CI*
**Fixed effects (IRR** † **)**				
Early cases (yes *vs.* no)			6.801	(4.004,10.910)
Neighbouring HCCAs with MM A cases‡ (%)			2.365	(2.078,2.695)
Road (yes *vs.* no)			1.743	(1.173,2.474)
Early rains (yes *vs.* no)			0.353	(0.239,0.502)
Mean seasonal humidity‡ (%)			0.656	(0.405,0.949)
**Random effects**				
Spatial structured hyperparameter (*σ_u_^2^*)	1.470	(0.799,2.295)	0.174	(0.010,0.488)
Spatial unstructured hyperparameter (*σ_v_^2^*)	1.965	(1.278,2.761)	2.579	(1.974,3.294)
Temporal hyperparameter (*σ_φ_^2^*)	1.755	(0.539,5.154)	0.303	(0.073,0.978)
**Overdispersion parameter** (*κ^−1^*)	4.009	(3.485,4.601)	2.586	(2.223,2.998)

Posterior mean parameter estimates and their 95% credible intervals (CIs) for the “null” model (no covariates included) and the multivariate model.

* CI: Bayesian credible interval.

† IRR: Incidence rate ratio.

‡ Standardized variables.

Regarding fixed effects, five covariates were significantly associated with MM A incidence (the 95% CI of their associated IRR did not contain 1). A reduced risk was associated with higher average relative humidity during the meningitis season (November–June) over the study region (posterior mean IRR = 0.656, 95% CI 0.405–0.949). Early rains in March in an HCCA represented a protective spatio-temporal factor (IRR = 0.353, 95% CI 0.239–0.502). The analyses identified three non-climatic factors; a positive association was found between disease incidence and percentage of neighbouring HCCAs having at least one MM A case during the same epidemiological year (IRR = 2.365, 95% CI 2.078–2.695), as well as presence of a road crossing the HCCA (IRR = 1.743, 95% CI 1.173–2.474) and occurrence of early cases before 31 December in a HCCA (IRR = 6.801, 95% CI 4.004–10.910).

The variances of the spatially structured and unstructured random effects were respectively 0.174 (95% CI 0.010–0.488) and 2.579 (95% CI 1.974–3.294) (Table 1). The posterior mean estimate of the spatial fraction was 0.062 (95% CI 0.004–0.166), meaning that most of the residual area-specific variability was spatially unstructured. Spatial correlation was almost totally captured by the multivariate model. The year-specific random effects also significantly contributed to the model (Table 1) and the inclusion of covariates helped to decrease the temporal random effects variance compared to the null model.

**Figure 3:**
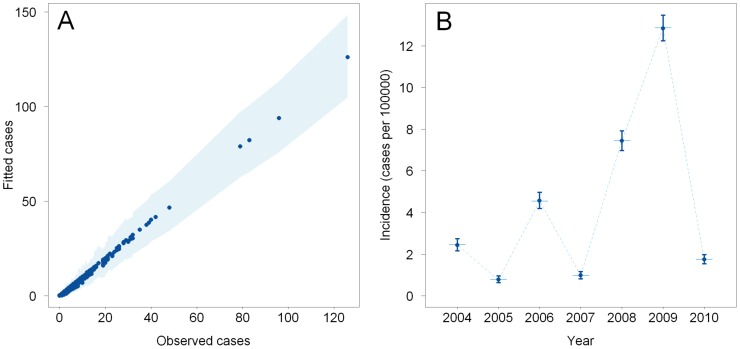
Multivariate model goodness of fit. (A) Scatter plot of the fitted posterior mean numbers of meningococcal meningitis A cases per year and health centre catchment area and their 95% credible intervals (CIs) (light-blue shaded region) versus the observed numbers. (B) Observed incidence rates (horizontal blue lines) per year over the study region and their corresponding fitted posterior mean annual incidence rates (filled dark-blue circles) and their 95%CIs (vertical dark-blue lines).

A scatter plot of the 4683 fitted posterior mean MM A cases versus the observed MM A cases shows the overall goodness of fit of the model (Figure 3.A). The inter-annual variations of incidence at the study region level were correctly captured by the model (Figure 3.B).

### Bayesian multivariate model over Tahoua subset

In Tahoua subset during 2002–2010, mass campaigns of A/C or A/C/W polysaccharide vaccination have been conducted in 53 HCCAs-years out of 665; the median reported vaccination coverage was 80.0% (IQR 53.5–89.2%).

The final multivariate model over Tahoua subset yielded similar results as the model over the whole study region (see Table S1). The same covariates were independently associated to disease incidence, except that early rains were no longer significant over this smaller geographical subset. No vaccination covariates were significant.

## Discussion

To our knowledge, this study is the first spatio-temporal statistical model in the African Meningitis Belt developed at a spatial scale as fine as the health centre catchment areas and using laboratory confirmed cases of meningococcal meningitis. Relying on advanced statistical methods, we demonstrated that both climatic and non-climatic factors (occurrence of early rains, mean relative humidity, occurrence of early cases, presence of roads and spatial neighbourhood interactions) are important for explaining spatio-temporal variations in MM A annual incidence at the HCCA level.

Appropriate statistical methods are necessary to investigate the underlying drivers of observed patterns of count data in small areas with spatio-temporal correlations. Hierarchical regression models of the Bayesian family have proven useful to analyse the spatio-temporal dynamics of infectious diseases in different settings, such as dengue in Brazil [29], soil-transmitted helminth infections in Kenya [30] or schistosomiasis in China [31]. The Bayesian formulation allows to acknowledge the uncertainty associated with all model (hyper)parameters (fixed and random), to include a spatial correlation structure within a prior distribution [32] and leads to more robust estimates in particular when the geographical level is small and the disease rare [33]. Such models are still rarely applied to MM in Africa. The modelling approach we adopted was a negative binomial GLMM using Bayesian estimation, to control for unobserved confounding factors and take into account the dependencies over space and time encountered in our dataset, incorporating year-specific and area-specific random effects [32], [34]. Ignoring these multiple correlations could lead to overestimate the significance of the covariates [31]. This model also accounted for extra-Poisson variation (overdispersion) in the count data via the negative binomial formulation, allowing the variance to be larger than the mean.

Another noteworthy feature of our analysis lies in its spatio-temporal resolution, uncommon for a country of the Belt. Since outbreaks have been shown to occur in spatially localized clusters at a sub-district level [4]–[6], we considered primordial to analyse MM A dynamics at a finer spatial scale than the more usual country or district levels [17]–[21], [35], [36]. From a public health perspective, the health centre catchment area used in this study is also a judicious choice. Indeed, the Nigerien health care system is based on these integrated health centres, which constitute the lowest health level (sub-district) and whose locations are chosen according to accessibility for populations. Regarding the time scale, to comply with our objective of explaining the overall annual burden of MM A in an area during each meningitis season, we chose to conduct analyses at the year level. We did not seek in this paper to model the seasonality of meningitis, which would have implied working at least at month (like in [22]) or week (like in [17], [18]) level. Our approach did not allow explaining intra-seasonal temporal dynamics and diffusion patterns – which were partly described in a previous paper [4]. This would constitute a distinct research question that the one tackled in this paper and should be further investigated.

The results of this study bring new insights into the epidemiology of MM in the Belt and the risk factors that play a role in the spatio-temporal variations of incidence. First, we observed that, at the study region level, higher annual incidence correlated with larger number of HCCAs having at least one MM A case, with larger number of localized outbreaks and, to a lesser extent, with higher intensity of these localized outbreaks. This brings support to Mueller and Gessner hypothesis that the magnitude of incidence during meningitis seasons in a region or country can increase if the geographical expansion and/or the intensity of localized epidemics increase [3]. The epidemiology thus changes from a regular year with a small number of localized epidemics in the region to an epidemic wave with many localized epidemics. We then sought to evaluate factors that could be associated with the occurrence of these localized incidence increases in a particular area during a particular year. Based on the factors that emerged from our model and that we discuss below, we hypothesize that spatio-temporal variations in MM A incidence between years and HCCAs result from variations in the intensity or duration of the dry season climatic effects on disease risk, and is further impacted by factors of spatial contacts, representing facilitated pathogen transmission.

First, the presence of primary roads and neighbourhood effects in the multivariate model indicates that human contacts and movements are important contributing factors that we assume to likely play a role in the transmission of the meningococcus, and/or an epidemic co-factor (e.g. respiratory virus [3]). HCCAs crossed by a road would be statistically more prone to re-infections from distant areas than isolated HCCAs outside the primary road network, and would experience higher transmission levels due to higher intensity of human movements and contacts. Yet, we cannot exclude that differences in accessibility to health centres contributed to bring out primary roads as a risk factor. One could also argue that health centres served by a road sent more CSF samples due to easier logistics. However, another study conducted in Niger and based on reported suspected cases (not affected by a potential logistic bias) also showed fewer absences and higher reappearance rates of meningitis cases in districts along primary roads [36]. The percentage of neighbours with cases, representing local spatio-temporal interactions, is not a surprising risk factor. Indeed, a previous study [4] showed that cases usually tended to be clustered in space and that these clusters most often encompassed a small number of HCCAs. Areas with more infected neighbours would be more likely to be infected by local spatial transmission.

Then, the presence of climatic parameters in the multivariate model indicates that, beyond an influence on MM seasonality agreed by several authors [2], [3], [17]–[19], climate can have a quantitative impact on inter-annual variations of incidence. The main physiopathological hypothesis for the role of climate is that dryness and dusty winds would damage the nasopharyngeal mucosa and increase the risk of bloodstream invasion by a colonizing meningococcus, and thus the case-to-carrier ratio [2]. Here, we found that annual incidence was negatively correlated to mean seasonal humidity over the study region. This factor was purely temporal (equal IRR for all spatial units within the same year), suggesting that humidity did not have spatial, but only temporal effect. At the study region level, the seasons of highest MM A incidence were also the seasons of lowest mean humidity. The between-year variations in humidity were not large but the results suggest that even a small decrease in humidity, resulting in a small increase in the case-to-carrier ratio according to the physiopathological hypothesis, can have a significant impact on the global MM A risk in all HCCAs, as these dryer conditions start in October and persist over several months (cumulative effect) and over a large geographical region. Similarly, Yaka et al detected a quantitative effect of climate on inter-annual variations of meningitis at the country level but November and December northerly winds were their best predictors [20]. This difference might be explained by the fact that they only considered the climatic conditions over the early dry season and not over the whole meningitis season. Interestingly, a second climatic factor, the occurrence of early rains in March, has a significant effect at the HCCA level. It has been noticed that the meningitis season seemed to stop at the onset of the rainy season, again explained by a decrease in invasiveness possibly due to less irritating conditions for the pharyngeal mucosa [2]. Our results are in agreement with this observation and, more precisely, show that the local occurrence of first rains in March, i.e. before the real beginning of the rainy season in the country, is a protective factor. The rains would thus stop the harmful effect of dryness and prevent local outbreaks to further develop.

The last and particularly strong factor that emerged from our model is the presence of early cases in a HCCA (before 31 December). It can be interpreted as a risk factor in itself (an outbreak would have more time to develop if it starts earlier), as an indicator of longer exposure to irritating climatic conditions of the dry season, or as a proxy of other factors responsible for the presence of the bacteria and higher levels of carriage and/or invasion. In any case, this parameter remains a strong determinant of high incidence in a HCCA. At the study region level, we also showed that the annual MM A incidence was correlated to the number of HCCAs with at least one early case and to the overall early incidence. Two other studies stressed the importance of early cases in the final size of the epidemic: an early onset was a good predictor of an epidemic at the district level in [37] and the number of cases during the peak months increased with the number of early cases occurring between October and December at the country level (Niger) in [20]. WHO also considers early cases in the season as a warning sign of large epidemic [11].

Surprisingly, vaccination the previous or the two previous year(s) was not found to be a protective factor in Tahoua subset. However, we cannot rule out the possibility that the low number of vaccinated HCCAs-years in our subset (8%) may have induced a lack of power to show a true protective effect of vaccination. This result could also be partially due to the decline of polysaccharide vaccine efficacy to 87% and 70% at one and two years after vaccination, respectively [38]. It is also possible that the provided data lack representativeness and over-estimate the real coverage. Of note, we decided not to study the impact of year *n* vaccination on year *n* incidence within this model formulation, as reactive vaccination would be associated with larger outbreaks (those which required vaccination) and, considering delays in implementing vaccination campaigns, would artificially appear as a risk factor in the model [39], [40].

Residual spatio-temporal variations that remained unexplained by the covariates included in our model suggest that other unknown or unmeasured factors contributed to the observed incidence. First, because our study concerned an ecologic investigation, suspected factors at the individual level (e.g. age, immuno-depression, smoking…) could not be accounted for. Then, the temporal variations at the country level could be suspected to be influenced by higher susceptibility due to waning pre-existing immunity [15] or emergence of a new variant that can escape herd immunity [13], [41]. However, the length of the study period did not enable us to study these effects: molecular characterization of *Nm* A isolates showed that the same sequence type (ST-7) was predominantly circulating in Niger during 2004–2010 [42], [43]. At the spatial level, the residual purely spatial variation observed in our model was mainly unstructured. The covariates better explained the spatial correlation, which both reflects shared environmental conditions and true epidemic diffusion, than the unstructured spatial variations. This suggests that other factors specific to each HCCA, such as quality of local health services or local behavioural practices, could contribute to explain the between-area heterogeneity in MM A incidence. The difficulty to measure such factors made the inclusion of area-level random effects necessary. Finally, other unexplained factors, such as respiratory viral co-infections, might contribute to the residual spatio-temporal heterogeneity, via an effect on transmission, colonization and/or invasion [3]. Although difficult to collect retrospectively, these factors should be further investigated at the health centre level and at least properly accounted for in any modelling attempt. Mathematical models, still little developed on this topic [44], could also help us to better understand the role of carriage and immunity in the epidemic dynamics.

This study relied on a unique dataset which provided a very precise picture of MM A spatio-temporal dynamics in Niger over seven years, and has already been used in published spatio-temporal analyses [4], [5]. The cases were all biologically confirmed by CERMES laboratory, which allowed us to exclude misclassified infectious agents that give similar clinical signs of meningitis. Databases commonly used by most statistical studies on MM in the Belt (e.g. [17]–[21]) gather clinically-suspected cases of meningitis, and thus include a mixture of different *Nm* serogroups and other bacteria such as *S. pneumoniae* and *H. influenzae*. In Niger, over our study period, 40% of positive CSFs were infected by another agent that *Nm* A. Relying only on suspected cases would therefore introduce a large number of misclassified cases. If etiological confirmation by conventional PCR may have led to under-estimation of positive cases [45], this should have affected the data in a spatially and temporally consistent way, as biological testing was performed with the same PCR assay by the same laboratory all along the study period. Our system may also suffer from underreporting from areas where performing a lumbar puncture and shipping the samples may represent logistical difficulties. We therefore excluded from the analyses the remote northern regions where population is very sparse and mainly nomadic. We can also consider that the potential variability in reporting rates has been taken into account through the explicit inclusion of overdispersion and spatial heterogeneity in our model. Besides, this surveillance system was extended to the whole country in 2002 (it was only effective in the capital city before) and might have been unsteady during the first months following its implementation. To reduce this potential temporal bias, we started our analyses in 2004, being confident that the system had thus reached a stable state. Finally, like in many other settings, the population affected by meningitis may not be entirely covered by the surveillance system. However, we can reasonably assume that most meningitis cases, because of their severity, end up reaching the health centres, with or without prior self-treatment or consultation of a tradi-practitioner [46]. Moreover, social and spatial disparities in care-seeking behaviours are probably reduced by free healthcare offered to all people suffering from meningitis in Niger. For all the reasons above, we are thus confident that the surveillance system is representative enough and that underreporting did not substantially affect the validity of our results, which are more likely to reflect the true underlying risk factors than the spatial disparities in the surveillance system efficiency.

To conclude, this study brings new insights into the epidemiology of meningitis in the Belt and allowed us to disentangle the climatic and non-climatic risk factors that play a role in the spatio-temporal variations of annual incidence at the health centre level. Besides, in the light of our results, a potential predictive model could rely on factors such as early cases in an area and its neighbours and early climatic conditions, provided their predictive value is evaluated. This could aid the development of an early warning system at the beginning of the meningitis season, following other recent attempts [47]. Despite new hope brought by the introduction of a meningococcal A conjugate vaccine [48], the ways in which the meningococcus will adapt to this changing situation are unknown and other serogroups such as W and X might replace A as the dominant serogroup. Such modelling could thus be tested on these serogroups, which would likely be influenced by most of the identified risk factors due to similar ways of transmission and invasion, and applied in other sub-Saharan countries sharing these peculiar epidemiological and climatic features.

## Supporting Information

Figure S1 **Seasonality of climate and aerosols.** Annual cycles of daily meteorological variables (temperature, relative humidity, precipitation, U wind and V wind) and monthly Absorbing Aerosol Index (AAI) averaged over the study region between 2004 and 2010.
(TIF)

Figure S2 **Epidemiological characteristics related to annual incidence.** Correlation between the annual meningococcal meningitis (MM) A incidence in the study region and epidemiological features of the MM A cases distribution (annual in dark blue and early in light blue) in health centre catchment areas (HCCAs) over the seven years of the study period.
(TIF)

Table S1 **Tahoua model results.** Results from the Bayesian hierarchical model of meningococcal meningitis (MM) A annual incidence at the health centre catchment area (HCCA) level over Tahoua subset, Niger 2004–2010: Posterior mean parameter estimates and their 95% credible intervals (CIs) for the “null” model (no covariates included) and the multivariate model.
(DOC)

Text S1 **Explanations on the computation of climate and aerosol covariates based on the seasonal cycles shown in Figure S1.**
(DOC)

Text S2 **Model description.**
(DOC)
