# Supplementary figures and images for: Spatio-Temporal Factors Associated with Meningococcal Meningitis Annual Incidence at the Health Centre Level in Niger, 2004–2010
Source: PLoS Negl Trop Dis. 2014 May 22;8(5):e2899. doi: 10.1371/journal.pntd.0002899 (PMC4031065; doi:10.1371/journal.pntd.0002899)

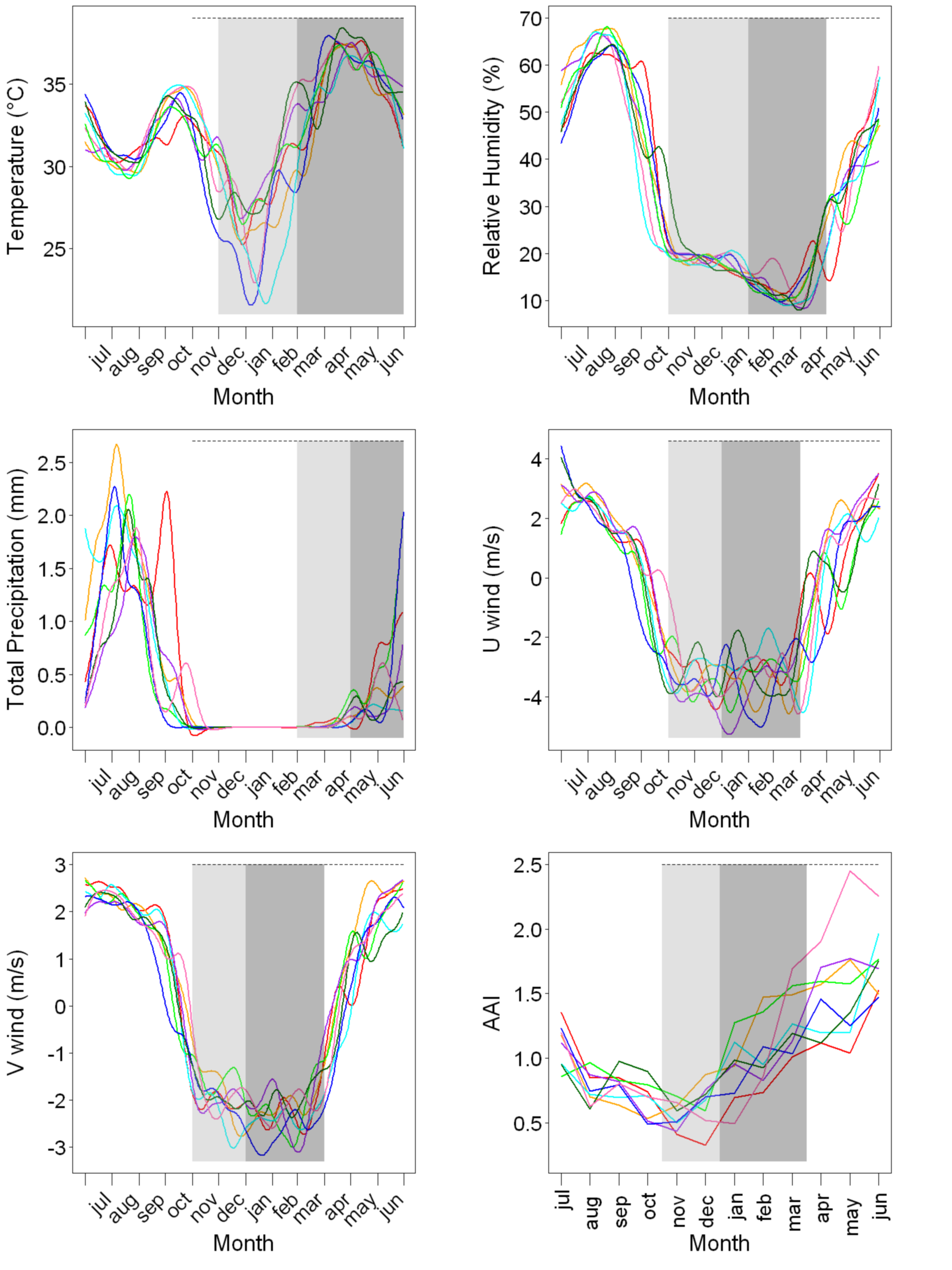

Supplement: Figure S1 — Seasonality of climate and aerosols. Annual cycles of daily meteorological variables (temperature, relative humidity, precipitation, U wind and V wind) and monthly Absorbing Aerosol Index (AAI) averaged over the study region between 2004 and 2010. (TIF) [file pntd.0002899.s001.tif]

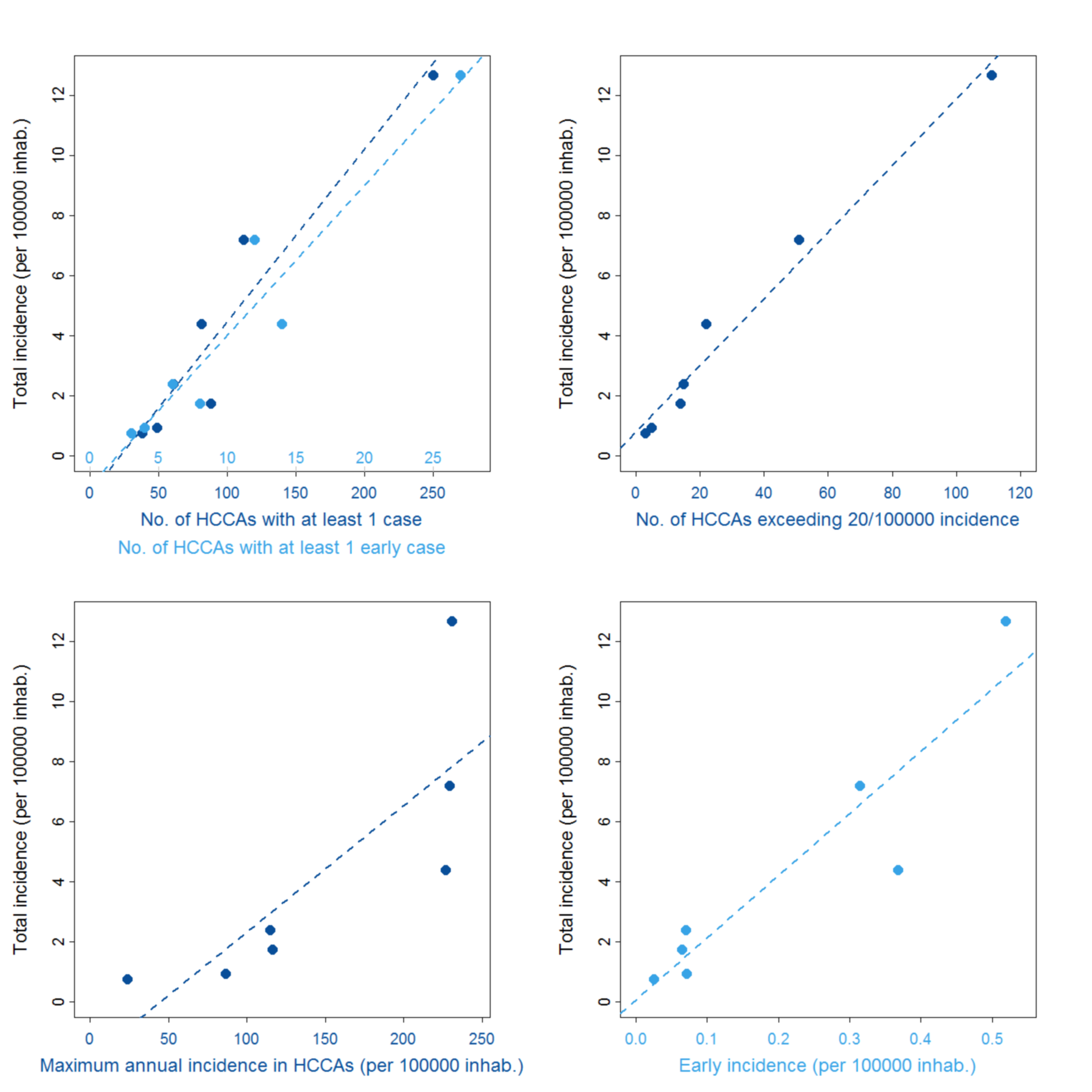

Supplement: Figure S2 — Epidemiological characteristics related to annual incidence. Correlation between the annual meningococcal meningitis (MM) A incidence in the study region and epidemiological features of the MM A cases distribution (annual in dark blue and early in light blue) in health centre catchment areas (HCCAs) over the seven years of the study period. (TIF) [file pntd.0002899.s002.tif]
