# Supplementary material for: Spatio-Temporal Factors Associated with Meningococcal Meningitis Annual Incidence at the Health Centre Level in Niger, 2004–2010
Source: PLoS Negl Trop Dis. 2014 May 22;8(5):e2899. doi: 10.1371/journal.pntd.0002899 (PMC4031065; doi:10.1371/journal.pntd.0002899)
Supplement: Table S1 — Tahoua model results. Results from the Bayesian hierarchical model of meningococcal meningitis (MM) A annual incidence at the health centre catchment area (HCCA) level over Tahoua subset, Niger 2004–2010: Posterior mean parameter estimates and their 95% credible intervals (CIs) for the “null” model (no covariates included) and the multivariate model. (DOC) [file pntd.0002899.s003.doc]

|  | **Tahoua null model** | | **Tahoua multivariate model** | |
| --- | --- | --- | --- | --- |
| **Parameters** | **Posterior mean** | **95% CI *** | **Posterior mean** | **95% CI *** |
| **Fixed effects (IRR †)** |  |  |  |  |
| Early cases (yes *vs.* no) |  |  | 6.087 | (2.113,14.920) |
| Neighbouring HCCAs with MM A cases ‡ (%) |  |  | 2.834 | (1.845,4.319) |
| Road (yes *vs.* no) |  |  | 2.471 | (1.067,5.000) |
| Mean seasonal humidity ‡ (%) |  |  | 0.530 | (0.276,0.845) |
| **Random effects** |  |  |  |  |
| Spatial structured hyperparameter (*σ_u_²*) | 0.110 | (0.001,0.905) | 0.022 | (0.001,0.142) |
| Spatial unstructured hyperparameter (*σ_v_²*) | 1.539 | (0.504,2.886) | 1.842 | (0.833,3.292) |
| Temporal hyperparameter (*σ_φ_²*) | 3.327 | (1.016,9.495) | 0.324 | (0.001,1.317) |
| **Overdispersion parameter** (*κ ^-1^*) | 2.285 | (1.623,3.133) | 1.830 | (1.257,2.562) |
| * CI : Bayesian credible interval |  |  |  |  |
| † IRR : Incidence rate ratio |  |  |  |  |
| ‡ Standardized variables |  |  |  |  |
