# Supplementary material for: Spatio-Temporal Factors Associated with Meningococcal Meningitis Annual Incidence at the Health Centre Level in Niger, 2004–2010
Source: PLoS Negl Trop Dis. 2014 May 22;8(5):e2899. doi: 10.1371/journal.pntd.0002899 (PMC4031065; doi:10.1371/journal.pntd.0002899)
Supplement: Text S1 — Explanations on the computation of climate and aerosol covariates based on the seasonal cycles shown in Figure S1. (DOC) [file pntd.0002899.s004.doc]

The study region has a semi-arid tropical climate, characterized by two main seasons: a rainy season from June to September and a long dry season that can be subdivided into a hot season (October to mid-November), a cooler season (end of November to February) and a very hot season (March to May). The seasons are caused by the annual north-south movement of the intertropical convergence zone: during the wet season, rains are brought by the monsoon winds blowing from the southwest whereas the dry and dust-laden Harmattan winds blow from the Sahara during the dry season. As we were interested in how the climate of a given year or season can influence the annual epidemic magnitude, we calculated multi-monthly means of each climatic variable to include in the model, averaged over periods relevant to the meningitis season or the seasonal cycles of each climatic variable, both for each health centre catchment area and for the whole study region. First, each climatic variable was averaged over the broad meningitis season (November to June) represented on Figure S1 by a horizontal dotted line at the top of each panel. In addition, sub-periods within the meningitis season were chosen according to the seasonal cycles of each climatic variable. These periods are represented by grey shading on Figure S1 and were chosen as follows. For temperature, two key periods were identified: December-February (cooler season) and March-June (very hot season). For relative humidity, two key periods were identified: November-January (first half of the very dry season) and February-April (second half of the very dry season). For precipitation, two key periods were identified: March or March-April (early rainfalls), and May-June (very beginning of the rainy season). For the last three variables, zonal wind component (U), meridional wind component (V) and AAI, two key periods were identified: November-December (first half of the Harmattan season) and January-March (second half of the Harmattan season). After March, the aerosols rise into the atmosphere and the AAI is no longer representative of the dust at the surface [17].The months of April/May represent a transition period between the Harmattan and the monsoon regimes.
