# Supplementary material for: Spatio-Temporal Factors Associated with Meningococcal Meningitis Annual Incidence at the Health Centre Level in Niger, 2004–2010
Source: PLoS Negl Trop Dis. 2014 May 22;8(5):e2899. doi: 10.1371/journal.pntd.0002899 (PMC4031065; doi:10.1371/journal.pntd.0002899)
Supplement: Text S2 — Model description. (DOC) [file pntd.0002899.s005.doc]

Basic modelling framework

The modelling approach we adopted was a negative binomial generalized linear mixed model (GLMM). GLMMs combine the properties of two statistical frameworks, linear mixed models (which incorporate random effects on the linear predictor scale) and generalized linear models (which handle non-normal data by using link functions and exponential family) (Bolker 2009). Let *Y_it_* denote the number of observed MM A cases in HCCA *i* (*i* = 1,…,669) and year *t* (*t* = 1,…,7). We assumed that the counts *Y_it_* followed a negative binomial distribution with an unknown scale parameter *κ* and mean *μ_it_=λ_it_E_it_*, where *E_it_* was the expected number of cases in HCCA *i* and year *t* (calculated as the population of HCCA *i* and year *t* multiplied by the overall MM A incidence rate for the whole dataset) and *λ_it_* was the unknown relative risk for HCCA *i* and year *t*. We specified a log-linear model for the relative risks as a function of covariates and appropriate spatio-temporal random effects.

We included random effects at the HCCA-level (spatial random effects) and the year level (temporal random effects) to account for unknown or unmeasured risk factors by introducing an extra source of variability into the model. The HCCA-level random effects *V_i_* captured the influence of factors that were specific to each area and that made observations from the same HCCA more likely to be alike (e.g. quality of local health services or local behavioural practices). Similarly, we included yearly random intercepts *φ_t_* to adjust on factors that influenced the overall annual epidemic risk (e.g. arrival of a new strain or waning herd immunity) and made observations from the same year more likely to be alike. We also tested a spatio-temporal interaction term *θ_it_* (observation-level random effects). Alternative sub-models were tested in R software within the frequentist framework, estimating parameters by maximum likelihood, and compared using Akaike Information Criterion (AIC) to determine the hierarchical structure that led to the best balance between goodness of fit and complexity. The resulting AICs are given in the table below. The model with the lowest AIC included the spatial and temporal random effects.

Afterwards, we used exploratory univariate analyses to identify covariates *X_k_* significantly associated with MM A incidence at 5% level by likelihood ratio test. We then performed a forward stepwise selection procedure based on AIC, manually incorporating the selected covariates, until no additional covariate improved the model’s AIC. We checked interactions and collinearity between covariates.

**Table of AICs and components of submodels (*α* is the overall intercept)**

| **Submodel** | **AIC** | **Model for *log(λ_it_)*** |
| --- | --- | --- |
| **1** | 6859.1 | $\alpha$ |
| **2** | 6760.5 | $\alpha+V_{i}$ |
| **3** | 6594.4 | $\alpha+\phi_{t}$ |
| **4** | 6350.2 | $\alpha+V_{i}+\phi_{t}$ |
| **5** | 6350.0 | $\alpha+V_{i}+\phi_{t}+\theta_{it}$ |
| **6** | 5949.1 |  |

Bayesian formulation and implementation

Finally, the best model was estimated in a Bayesian framework, which leads to more robust estimates when the geographical level is small and the disease rare [33] and allowed us to further include spatial and temporal correlation structures within prior distributions [32]. Within this framework, we thus decomposed the HCCA-level random effect into two components, following the model proposed by Besag et al (1991), known as the convolution BYM (Besag, York and Mollié) model. To the previous unstructured spatial random part *V_i_*, we added a second component, which captured the influence of spatially correlated effects *U_i_* that made observations which occurred closer together in space more likely to be alike (e.g. other environmental effects). Similarly, we also tested structured temporal random effects *θ_t_* (as random walk of order 1). These structured temporal effects did not improve the model’s Deviance Information Criterion (DIC), but the BYM formulation lowered the DIC of 17 units compared to the model with a single unstructured spatial component. The final formulation of our Bayesian GLMM was then:

$Y_{it}$~$NegBin(\mu_{it}=\lambda_{it}E_{it},\kappa)$ $i=1,...669,t=1,...,7$

$$\log(\mu_{it})=\log(E_{it})+\log(\lambda_{it})$$

$\alpha$~$U(-\infty,+\infty)$

$\beta_{k}$~$N(0,10^{5})$

$U_{i}$~$CAR(W,\sigma_{u}^{2})$

$V_{i}$~$N(0,\sigma_{v}^{2})$

$\phi_{t}$~$N(0,\sigma_{\phi}^{2})$

$\tau_{u}=\frac{1}{\sigma_{u}^{2}}$, $\tau_{v}=\frac{1}{\sigma_{v}^{2}}$, $\tau_{\phi}=\frac{1}{\sigma_{\phi}^{2}}$ ~$Ga(0.5,0.0005)$

$\log(\kappa)$~$N(0,10^{4})$

The term *α* was the overall intercept. The *X_k_* represented the spatio-temporal covariates and the *β_k_* the associated regression coefficients. The spatially and temporally unstructured random effects were assigned normal priors. The spatially structured random effects were assigned a conditional autoregressive (CAR) model prior, as follows:

$U_{i}\left|U_{j,j\neq i}\right.$~$N(u_{i},\sigma_{i}^{2})$, $u_{i}=\frac{\sum_{j} w_{ij}U_{j}}{\sum_{j} w_{ij}}$, $\sigma_{i}^{2}=\frac{\sigma_{u}^{2}}{\sum_{j} w_{ij}}$

where the weights *w_ij_* reflected the spatial proximity of HCCA *i* and *j*. We considered an adjacency neighbourhood structure where *w_ij_* = 1 if HCCAs *i* and *j* shared a common boundary and *w_ij_* = 0 otherwise. Only first order neighbours were considered since a previous analysis showed that the median size of spatial clusters was of two neighbouring HCCAs (Paireau 2012). This CAR model implied that each *U_i_* conditional on the neighbouring *U_j_* followed a normal distribution with mean equal to the average of neighbouring *U_j_* and variance inversely proportional to the number of neighbours *n_i_ =* Σ*_j_ w_ij_*. As the CAR model formulated above was improper, a uniform flat prior for the intercept *α* was specified and a sum-to-zero constraint was imposed on the *U_i_* (Best 1999). The relative contribution of spatial *vs* unstructured heterogeneity was given by the spatial fraction, defined as the marginal variance of the spatial effects divided by the sum of this variance with the marginal variance of the unstructured random effects. Because we wanted the observations, not the priors, to drive the conclusions, we chose vague priors. We chose diffuse Normal priors with mean 0 and precision 1x10^-5^ for the regression coefficients *β_k_*. Gamma hyperprior distributions with shape parameters 0.5 and inverse scale parameters 0.0005 were specified for the inverse variances (precisions) of the spatial and temporal random effects, following Kelsall and Wakefield (1999). We used a Normal prior with mean 0 and precision 1x10^-4^ for the log scale parameter of the negative binomial distribution.

The model was fitted in the Bayesian framework using Markov Chain Monte Carlo (MCMC) simulation methods implemented in WinBUGS software. Because models must be completely specified by the user, WinBUGS may not be viewed by some as being as user-friendly as other statistical software packages, but having to specify a model in WinBUGS insures a basic understanding of underlying processes that may not accompany use of many common statistical software packages. Model code is available from the corresponding author. We standardized the relevant covariates to zero mean and unit variance, to aid MCMC convergence. Two parallel MCMC chains were run starting from different initial values. Convergence was assessed by visual examination of time-series plots and calculation of Gelman&Rubin diagnostic test (Gelman and Rubin 1992). On this basis, each chain was run for 200000 iterations, discarding the first 25000 samples of each simulation as burn-in, with a thinning of 5 (to reduce autocorrelation). Posterior estimates were based on pooling the 2x35000 samples, giving Monte Carlo standard errors of less than 5% of the posterior standard deviation for all parameters, as usually recommended (Roberts 1996). For epidemiological interpretation, we exponentiated the regression coefficients (*exp(β_k_)*) to represent the incidence rate ratios (IRRs) associated with a unit increase in exposure (*i.e.* the incidence rate for a given value (respectively category) of a continuous (resp. categorical) exposure variable, divided by the incidence rate for that exposure value (resp. category) minus one unit (resp. level)), and calculated their 95% credible intervals (CIs). We used a similar model formulation for the second model over Tahoua subset and followed the exact same procedure as described above. The only difference was the inclusion of vaccination data (described in the main Methods section) in the covariates selection process.

References

Besag J, York J, Mollié A (1991) Bayesian image restoration, with two applications in spatial statistics. Ann Inst Stat Math 43: 1–20.

Best N, Arnold R, Thomas A, Waller L, Conlon E (1999) Bayesian models for spatially correlated disease and exposure data. Bayesian Statistics 6:131–156.

Bolker BM, Brooks ME, Clark CJ, Geange SW, Poulsen JR, et al. (2009) Generalized linear mixed models: a practical guide for ecology and evolution. Trends Ecol Evol 24: 127–135.

Kelsall JE, Wakefield JC (1999) Discussion of “Bayesian models for spatially correlated disease and exposure data” by Best N, Arnold R, Thomas A, Waller L, Conlon E. Bayesian Statistics 6:131–156.

Gelman A, Rubin DB (1992) Inference from iterative simulation using multiple sequences. Statistical Science 7(4):457-472.

Paireau J, Girond F, Collard J-M, Maïnassara HB, Jusot J-F (2012) Analysing spatio-temporal clustering of meningococcal meningitis outbreaks in Niger reveals opportunities for improved disease control. PLoS Negl Trop Dis 6: e1577.

Roberts G (1996) Markov Chain Concepts Related to Sampling Algorithms. Markov Chain Monte Carlo in Practice (Gilks WR, Spiegelhalter DJ, Richardson S, eds). London: Chapman and Hall.
